# Omni‐functional crystal: Advanced methods to characterize the composition and homogeneity of lithium niobate melts and crystals

**DOI:** 10.1002/EXP.20220059

**Published:** 2022-08-16

**Authors:** Kunfeng Chen, Ji'an Wu, Qianyu Hu, Zheng Lu, Xiangfei Sun, Zhiqiang Wang, Gongbin Tang, Hui Hu, Dongfeng Xue

**Affiliations:** ^1^ Institute of Novel Semiconductors State Key Laboratory of Crystal Materials Shandong University Jinan China; ^2^ School of Physics State Key Laboratory of Crystal Materials Shandong University Jinan China; ^3^ Multiscale Crystal Materials Research Center Shenzhen Institute of Advanced Technology Chinese Academy of Sciences Shenzhen China

**Keywords:** characterization, composition homogeneity, lithium niobate, microstructure

## Abstract

Lithium niobate (LN) is a type of multifunctional dielectric and ferroelectric crystal that is widely used in acoustic, optical, and optoelectronic devices. The performance of pure and doped LN strongly depends on various factors, including its composition, microstructure, defects, domain, and homogeneity. The structure and composition homogeneity can affect both the chemical and physical properties of LN crystals, including their density, Curie temperature, refractive index, and piezoelectric and mechanical properties. In terms of practical demands, both the composition and microstructure characterizations these crystals must range from the nanometer scale up to the millimeter and wafer scales. Therefore, LN crystals require different characterization technologies when verifying their quality for various device applications. Optical, electrical, and acoustic technologies have been developed, including x‐ray diffraction, Raman spectroscopy, electron microscopy, and interferometry. To obtain detailed structural information, advanced sub‐nanometer technologies are required. For general industrial demands, fast and non‐destructive technologies are preferable. This review outlines the advanced methods used to characterize both the composition and homogeneity of LN melts and crystals from the micro‐ to wafer scale.

## Graphical abstract

For optical, electrical, and acoustic device applications, the advanced characterization method for measuring composition, microstructure, defect, domain, homogeneity, Curie temperature, refractive index, piezoelectricity of LiNbO_3_ bulk crystal is needed.

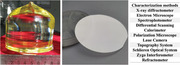

## 1 INTRODUCTION

Designed as special multifunctional materials, lithium niobate (LN) crystals have attractive electro‐optic, acousto‐optic, piezoelectric, and nonlinear optical properties, which have been widely exploited in surface acoustic wave filters, optical waveguides, waveguide lasers, polarizers for optic isolators, and holographic storage.^[^ ^1^, ^2^ ^]^ In 2017, researchers at Harvard University announced that it was entering “Lithium Niobate Valley,” because LN crystals could represent for optics what silicon was for electronics.^[^ ^3^ ^]^ The criteria for achieving this is the ability to fabricate optical microstructures using LN single crystal thin films such as those provided by the company NANOLN; furthermore, these optical microstructures can be used to fabricate integrated photonic, quantum‐photonic, and microwave‐to‐optical conversion devices.^[^ ^4^ ^]^ For device applications, bulk LN single crystals are in high demand; these are generally grown from melt via the Czochralski pulling method. The physical and chemical properties of LN single crystals are largely determined by their stoichiometry (Li/Nb ratio), point defects, dopant content, and domain structures.^[^ ^5^ ^]^ Li‐poor LN crystals exhibit point defects of both Li vacancies and Nb antisites, leading to Li/Nb cation mixing and disorders in the LN lattice frame.^[^ ^6^ ^]^ According to the Li_2_O‐Nb_2_O_5_ phase diagram, congruent LN (CLN) crystals can be readily grown from corresponding congruent melts (48.6 mol% Li). In this case, near‐stoichiometric LN (50 mol% Li) crystals can often be grown via special methods such as vapor transport equilibration, K_2_O flux, and the double crucible method [using an extremely Li‐rich melt (58% Li_2_O)].^[^ ^7^ ^]^

**FIGURE 1:**
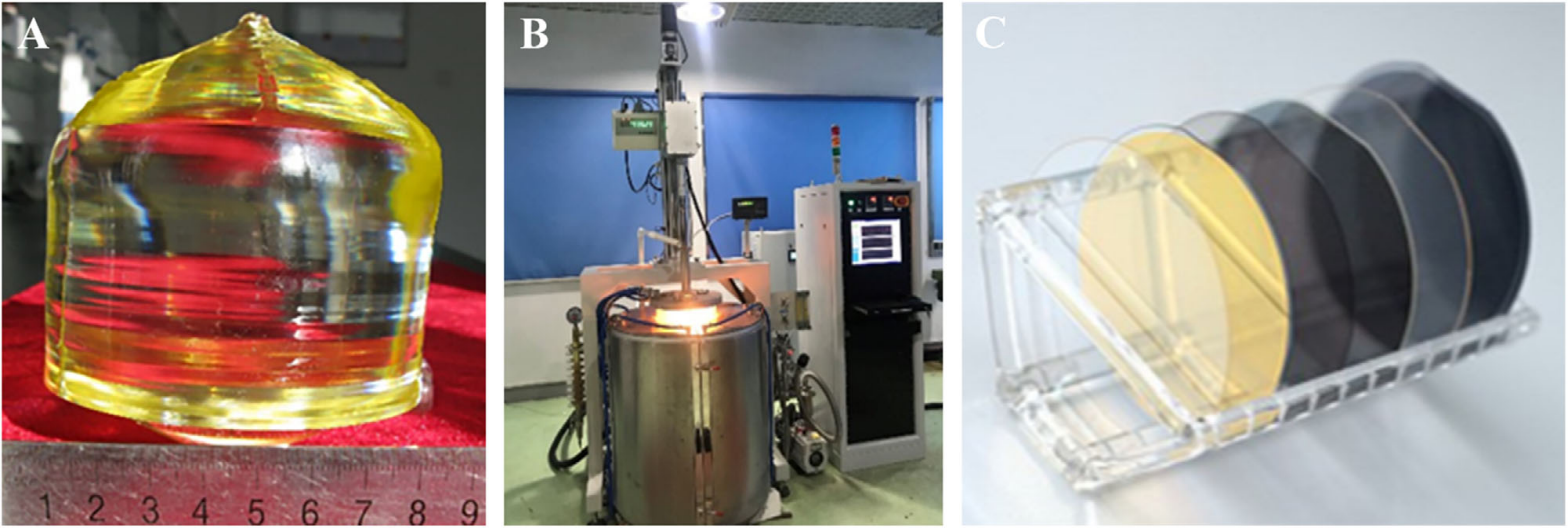
(A) ϕ4‐inch LN crystals grown via the Czochralski method in our lab. (B) Large‐size LN single crystal growth system. (C) 300–900 nm thickness LN single crystal thin films (NANOLN) from Jinan Jingzheng Electronics Co., Ltd.

Accurately measuring structural information and property data is vital in various practical applications of LN single crystals. LN wafers with different diameters (e.g., ϕ3–6 in) can be used for device manufacture; these are fabricated by cutting the LN bulk crystals (Figure 1A). Following the successful application of ion‐implantation technology (as achieved in our laboratory), LN single‐crystal thin films with thicknesses of 300–900 nm have been commercialized by our incubated NANOLN company (Figure 1C). LN single crystal thin films have been successfully applied to integrated modulators, acoustic filters, oscillators, and piezoelectric sensors.^[^ ^8^ ^]^ It should be clarified that implementing LN in bulk crystals, wafers, and devices typically requires various approaches when characterizing their corresponding physicochemical properties. In this review, we summarize some available characterization methods that have been applied to LN single crystals, wafers, and films.

## 2 PROPERTY EVALUATION AND MEASUREMENT METHODS FOR LN

Multifunctional LN crystals exhibit electro‐optic, acousto‐optic, piezoelectric, and nonlinear properties that stem from their unique crystallographic features. Typical data used to characterize the physical properties of LN crystals are available in numerous published studies. Table 1 lists several physical and optical data for CLN.^[^ ^9^, ^10^, ^11^, ^12^, ^13^ ^]^ For additional property data of LN crystals, please see refs. [9, 10, 11, 12, 13]. To satisfy device application requirements, it is necessary to accurately determine the LN property data. Table 2 lists several available characterization methods for evaluating LN single crystals and wafers. To accurately determine individual properties, different measurement methods are required. For example, when measuring the Curie temperature of LN, differential scanning calorimetry (DSC) and the dielectric constant method are often employed.

**TABLE 1:** Physical and optical properties of CLN single crystals.^[^ ^9^, ^10^, ^11^, ^12^, ^13^ ^]^ The data are Open Data, which are from ref. 9, 10, 11, 12, 13

Properties	Values
Crystal structure	Trigonal, space group *R*3*c*, point group 3*m*
Lattice parameters	*a* = 5.148 Å, *c* = 13.863 Å
Congruent melting point	∼1250°C
Curie temperature	∼1140°C
Mohs hardness	5
Density	4.64 g/cm^3^
Optical transmission	420–5200 nm
Refraction indices	*n*_e_ = 2.156, *n*_o_ = 2.232 @ 1064 nm *n*_e_ = 2.203, *n*_o_ = 2.286 @ 632.8 nm
Nonlinear optical coefficients (pm/V @ 1.06 μm)	*d*_22_ = 3, *d*_31_ = −5, *d*_33_ = −33
Specific heat (@ 25°C)	0.15 cal/g/°C
Thermal conductivity (@ 25°C)	10^−2^ cal/cm⋅sec °C
Thermal expansion (@ 25°C)	*α*_a_ = 15 × 10^−6^/°C *α*_c_ = 7.5 × 10^−6^/°C
Dielectric constants (@ 25°C)	Clamped (*υ* > 10 MHz): *ε*_11_ = 44, *ε*_33_ = 27.9
Electro‐optic coefficients (pm/V @ 633 nm)	Unclamped: *r*_13_ = 10, *r*_22_ = 7, *r*_33_ = 33, *r*_51_ = 33 *r*_z_ = 18
Pyroelectric coefficient (@ 25°C)	−4 × 10^−5^ C/°C/m^2^
Piezoelectric strain coefficients (@ 25°C and 10^−12^ C/N)	*d*_15_ = 69.2, *d*_31_ = −0.85 *d*_22_ = 20.8, *d*_33_ = 6.0
Elastic stiffness coefficients (constant field @ 25°C and 10^11^ N/m^2^)	C_11_ = 2.030, C_14_ = 0.085, C_44_ = 0.595, C_12_ = 0.573, C_33_ = 2.424, C_66_ = 0.728, C_13_ = 0.752

**TABLE 2:** Various characterization methods for LN single crystals

Characterization methods	Measured properties
X‐ray diffractometry	Crystallographic orientation
Electron microscopy	Microstructure
Spectrophotometry	Absorption spectrum
Differential scanning calorimetry	Melting point and Curie temperature
Polarization microscopy	Sub‐grains and crystal inclusion
Laue camera	Crystal orientation
Topography system	Distribution and pattern of wafer defects and strain [via two‐dimensional mapping (image)]
Schlieren optical system	Presence of sub‐grains and refractive index
Zygo interferometry	Wafer surface roughness and internal refractive index
Refractometry	Refractive index and birefringence for crystal block/wafer

LN crystal growth is closely related to the corresponding melt fluxion.^[^ ^14^ ^]^ Therefore, the quality of LN single crystals is strongly affected by their melt properties: composition, viscosity, density, and surface tension.^[^ ^14^, ^15^, ^16^ ^]^ Table 3 shows some physical and chemical properties of congruent LN melts; they are markedly temperature dependent. Thermal analysis technology has been used to confirm both the thermodynamic and kinetic behaviors of LN melts.^[^ ^16^ ^]^ Experimental studies have identified seven species in LN melts: LiNbO_3_, Li_2_O, Nb_2_O_5_, Li^+^, LiO^−^, Nb_2_O_4_V_O_^2+^, and O^2−^.^[^ ^16^ ^]^ It should be emphasized that for the study of high‐temperature LN melts, considerable work is still required, owing to the limitations of measurement technologies.

**TABLE 3:** Physical and chemical properties of congruent LN melts

Properties	Values
Density (@ 1533 K)	∼3.668 g/cm^3^
Thermal expansion coefficient	1.7 × 10^−4^/K
Surface tension (@ 1510 K)	317 × 10^−5^ N/cm
Capillary number	∼4.0 mm
Dynamical viscosity (@ 1560 K)	21.5 mPa⋅s (congruent melt) 18.9 mPa⋅s (stoichiometrical melt)
Specific heat (@ 1548 K)	0.23 cal/g/°C
Dissociation and ionization reaction	2LiNbO_3_ ↔ Li_2_O + Nb_2_O_5_ Li_2_O ↔ Li^+^ +LiO^−^ Nb_2_O_5_ ↔ Nb_2_O_4_V_O_^2+^ + O^2−^

## 3 COMPOSITION AND BONDING CHARACTERIZATION METHODS

Many physical properties of LN (e.g., Curie temperature and electro‐optic coefficients) strongly depend on its composition (i.e., the Li/Nb ratio). The phase diagram shows that congruent LN often exhibits an Li/Nb ratio of ∼48.6:51.4. Using the relationship between Li concentration and LN's physical properties, optical and thermal methods have been used to determine the Li concentration within LN crystals (Table 4).

**TABLE 4:** Different methods and corresponding equations used to determine Li concentration in LN crystals

Methods	Equation
Raman scattering method	*c*_Li_ = 53.03−0.4739Γ (E‐mode at 153 cm^−1^) *c*_Li_ = 53.29−0.1837Γ (A_1_‐mode at 876 cm^−1^)
UV absorption edge	*λ*_20_ = 320.4−1.829*c*_Li_−5.485*c*_Li_^2^ at 20 cm^−1^ *λ*_15_ = 321.9−1.597*c*_Li_−5.745*c*_Li_^2^ at 15 cm^−1^
Birefringence	*c*_Li_ = a(λ)+b(λ)Δ*n* Δ*n* = *n*_e_−*n*_o_
Curie temperature	*T*_c_ = 39.064*c*_Li_ − 746.73 *T*_c_ = 36.70*c*_Li_ − 637.30 *T*_c_ = 9095.2 − 369.05*c*_Li_ + 4.228*c* ^2^_Li_
Phase‐matching temperature	[Li_2_O] = 48.345 + 8.1543 × 10^–3^ *T*_pm_

### 3.1 Fourier transform infrared spectroscopy

Fourier transform infrared spectroscopy (FTIR) spectroscopy uses a Michelson interferometer to superpose two beams of complex infrared light whose optical range difference varies at a certain rate (thereby producing interferometric light) after interacting with the sample. The detector inputs the obtained interference signal into a computer for Fourier variation processing and converts the interferogram into a spectrogram. In LN determination, FTIR spectroscopy can be used to obtain the proton distribution on the surface of LN samples, as well as the corresponding molecular steric configuration.^[^ ^17^, ^18^ ^]^ In addition, it is also possible to determine the individual elemental components of LN samples according to the intensity of the absorption peaks in the spectra.^[^ ^19^ ^]^ In fact, hydrogen is present in almost all LN crystals that have not been specially treated after their growth process. Hydrogen ions enter the crystal lattice and combine with oxygen ions to form OH^−^. The position, waveform, peak width, and polarization characteristics of the OH^−^ vibrational absorption band relate to Li_2_O in the crystal. A close relationship was found between the content and concentration of doping ions in LN crystals. The OH^−^ absorption peak of the CLN crystal is located at ∼3484 cm^−1^, and the corresponding half‐peak width is ∼30 cm^−1^.^[^ ^20^ ^]^ When the concentration of Li^+^ in LN increases, the absorption band of OH^−^ is reduced, and the absorption of the high‐energy component decreases. When [Li]/[Nb] approaches the stoichiometric ratio, the OH^−^ infrared absorption spectrum becomes a single absorption peak with a line width of only 3 cm^−1^ and a peak at 3466 cm^−1^.^[^ ^21^ ^]^ LN defects decrease under the increase in Li_2_O content, causing the OH^−^ vibration mode to change to a single peak. Therefore, the [Li]/[Nb] ratio in the LN crystals can be qualitatively determined by measuring the OH^−^ infrared absorption spectrum.

### 3.2 Ultraviolet–visible spectroscopy

Ultraviolet–visible spectroscopy (UV–vis) spectroscopy measures the jumps of molecular valence electrons (or ions) after UV or visible light (10–800 nm) irradiation. Different structures exhibit different electron jumps (energies or wavelengths). For LN crystal determination, this technique can be used to qualitatively characterize the involved defects of LN samples,^[^ ^22^, ^23^ ^]^ to visualize the intensity of the absorption peaks for doped LN samples in relation to the various doping concentrations^[^ ^24^ ^]^ and obtain information related to the LN sample bandgap.^[^ ^25^, ^26^ ^]^ LN crystals are ferroelectric and feature an oxygen‐containing octahedral structure; their basic optical absorption edge can be determined from the charge transfer transition energy of oxygen 2*p* electrons to Nb^5+^ empty *d* orbitals. Therefore, changes in the electron cloud distribution of coordinated oxygen affect the position of the absorption edge. The ultraviolet absorption edge of stoichiometric LN crystals (i.e., wavelengths with an absorption coefficient α of 20 cm^−1^) is at ∼305.6 nm.^[^ ^27^ ^]^ Under an increase of Li_2_O content in LN crystals, the absorption edge corresponding to an absorption coefficient of 20 cm^−1^ is blue‐shifted from the 320 nm (denoting CLN crystals). Although the relationship between the LN crystal composition and the position of the absorption edge is nonlinear,^[^ ^28^, ^29^ ^]^ this remains a simple and effective method for determining the LN crystal composition by measuring the absorption edge of the LN crystal samples. The relationship between the UV absorption edge and Li_2_O content in LN crystals has been proposed as^[^ ^30^ ^]^

(1) $$\lambda_{20}=320.4-1.829x-5.485x^{2},$$

(2) $$\lambda_{15}=321.9-1.597x-5.745x^{2},$$

where* λ*_20_ and *λ*_15_ represent the wavelengths of the corresponding light waves when the absorption coefficients *α* are 20 and 15 cm^−1^. When the content of [Li_2_O] in the LN crystal exceeds 49.5%, the sensitivity of this method is particularly high; in this case, when the composition varies by 0.1 mol%, the position of the absorption edge shifts by 2 nm.^[^ ^31^, ^32^ ^]^

### 3.3 Curie temperature

The dielectric constants of LN crystals change abruptly near the ferroelectric–paraelectric phase transition temperature (i.e., the Curie temperature); therefore, the Curie temperature of LN crystals can be obtained by measuring the variation of the crystal's dielectric constant with respect to temperature. In addition to high‐temperature dielectric measurements for determining the LN crystal's Curie temperature, DSC can also be used to measure the heat flow at the ferroelectric–paraelectric phase transformation. The available research shows that the Curie temperatures of LN crystals are very sensitive to their composition, and the use of the Curie temperature to measure the corresponding crystal composition not only affords a high degree of accuracy but also proceeds via a relatively simple method.^[^ ^33^ ^]^ The relationship between the crystal composition and Curie temperature is given by Equations (3),^[^ ^31^ ^]^ (4),^[^ ^34^ ^]^ and (5):^[^ ^35^ ^]^

(3) $$T_{c}=39.064c-746.73,$$

(4) $$T_{c}=36.70c-637.30,$$

(5) $$T_{c}=9095.2-369.05c+4.228c^{2}.$$

### 3.4 Phase‐matching temperature

LN crystals are a type of negative uniaxial crystal that can realize a first‐type phase matching of o + o → e in the process of generating the second harmonic. Under an increase in [Li]/[Nb],^[^ ^36^ ^]^ the birefringence of the LN crystal increases, and the phase‐matching temperature also increases. Borcdui^[^ ^31^ ^]^ proposed a relationship between the phase‐matching temperature and Li_2_O concentration in LN crystals. However, when the [Li_2_O]/[Nb_2_O_5_] ratio in the LN crystal approaches 1, the [Li_2_O] content in the LN crystal does not vary linearly with the phase‐matching temperature *T*_pm_, and the above formula is no longer effective. Therefore, when the composition of the LN crystal is close to the stoichiometric ratio of Li/Nb, it is unsuitable to measure the [Li_2_O] content using the phase‐matching temperature method:

(6) $$\left[Li_{2}O\right]=48.345+8.1543\times 10^{-3}T_{\mathrm{pm}}.$$

### 3.5 Raman spectroscopy

The Raman spectroscopy studies of LN crystals show that when the [Li]/[Nb] ratio in the LN crystal increases, the vibrational frequency of individual vibrational modes of the LN crystal remains unchanged; however, the corresponding linewidth becomes significantly narrowed.^[^ ^37^, ^38^ ^]^ The half‐height width Γ of the two vibrational modes can be used to determine the Li_2_O content.^[^ ^6^, ^30^ ^,]^ At room temperature, LN is a ferroelectric crystal with a point group of *C*_3v_. According to group theory analysis and calculations, the optical vibration mode at the Γ point can be classified as 4A_1_ + 5A_2_ + 9E. The A_1_ and E modes are Raman‐IR active, and the A_2_ mode is non‐Raman and non‐IR active.^[^ ^39^ ^]^ The A_1_ and E modes can be distinguished by choosing different geometrical configurations. Table 5 lists the vibrational modes for the right‐angle scattering and backscattering (or forward scattering) geometric configurations.^[^ ^40^ ^]^

**TABLE 5:** Expected vibrational modes of LN based on selection rules. Reproduced with permission.^[^ ^40^ ^]^ Copyright 2015, AIP Publishing

Backscattering or forward‐scattering configuration	Modes	Right‐angle scattering configuration	Modes
$X\left(YY\right)\bar{X}$	A_1_(TO) + E(TO)	X(ZZ)Y	A_1_(TO)
$X\left(YZ\right)\bar{X}$	E(TO)	X(ZX)Y	E(TO + LO)
$X\left(ZY\right)\bar{X}$	E(TO)	X(YZ)Y	E(TO + LO)
$X\left(ZZ\right)\bar{X}$	A_1_(TO)	X(YX)Y	E(TO + LO)
$Y\left(XX\right)\bar{Y}$	A_1_(TO) + E(LO)	X(ZY)Z	E(TO)
$Y\left(XZ\right)\bar{Y}$	E(TO)	X(ZX)Z	Quasi‐modes
$Y\left(\mathrm{ZX}\right)\bar{Y}$	E(TO)	X(YY)Z	E(TO) + quasi‐modes
$Y\left(ZZ\right)\bar{Y}$	A_1_(TO)	X(YX)Z	Quasi‐modes
$Z\left(YX\right)\bar{Z}$	E(TO)	Z(XZ)Y	E(TO)
$Z\left(XY\right)\bar{Z}$	E(TO)	Z(YX)Y	E(TO)
$Z\left(XX\right)\bar{Z}$	A_1_(LO) + E(TO)	Z(YZ)Y	Quasi‐modes
$Z\left(YY\right)\bar{Z}$	A_1_(LO) + E(TO)	Z(XX)Y	Quasi‐modes

The position, width, and intensity of the peaks in the Raman spectra contain rich information regarding the chemical composition and structural features of LN. The lithium content in the LN crystal can be obtained by using linewidths Γ at ∼153 and 876 cm^−1^; the calculation formulas have been proposed as^[^ ^41^ ^]^

(7) $$c_{\mathrm{Li}}\left[\mathrm{mol}\%\right]=53.03-0.4739\;\Gamma \;\left[cm^{-1}\right]\mathrm{for}\mathrm{the}\;153\;cm^{-1}\mathrm{phonon},$$

(8) $$c_{\mathrm{Li}}\left[\mathrm{mol}\%\right]=53.29-0.1837\;\Gamma \;\left[cm^{-1}\right]\;\mathrm{for}\mathrm{the}\;876\;cm^{-1}\mathrm{phonon}.$$

The line width Γ can be obtained using Gaussian or Lorentzian fitting. The Raman scattering method is widely applicable, even when the sample size is small. If no single crystal is polished, the Raman technique is applied to a ball with a properly aligned but incomplete surface. In this case, the excitation laser beam strikes the surface during grazing incidence. The presence of stray light in the scattering process enhances the geometric baseline problem. Schlarb et al. suggested using only the 876 cm^−1^ mode.^[^ ^4^, ^5^ ^]^

Oswaldo et al.^[^ ^42^ ^]^ have reported the following formulas for determining niobium content in LN powder at ∼876 cm^−1^:

(9) $${\left(C_{\mathrm{Nb}}\right)}_{L}\left[\mathrm{mol}\%\right]=\left[256.4103\times \left(\Gamma_{L}/2x_{c}\right)+43.5385\right]\pm 0.4,$$

(10) $${\left(C_{\mathrm{Nb}}\right)}_{G}\left[\mathrm{mol}\%\right]=\left[588.2353\times \left(\Gamma_{G}/2x_{c}\right)+42.7059\right]\pm 0.5.$$

The Γ subscripts L and G denote Lorentzian and Gaussian fittings, respectively; here, x_c_ denotes the center of the Raman band.

**FIGURE 2:**
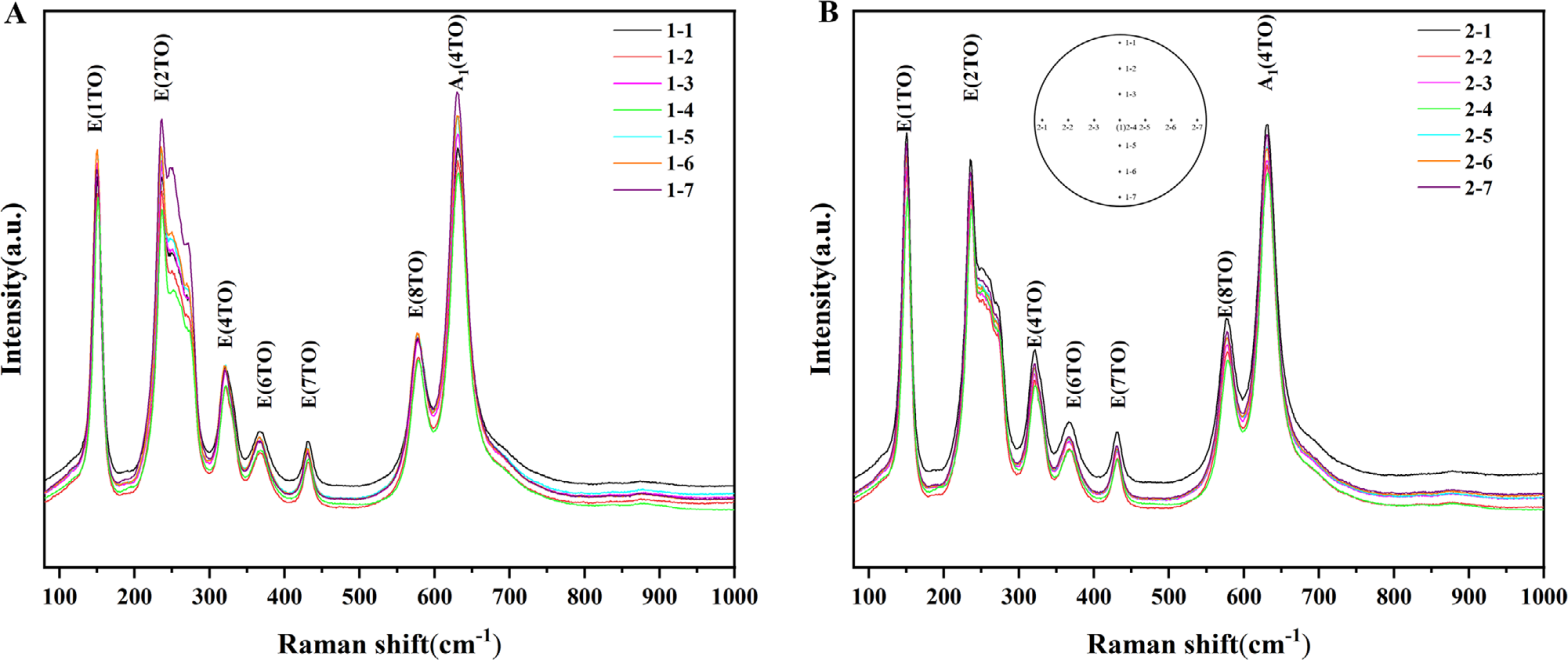
Raman spectra of LN wafer, as measured with a laser wavelength of 532 nm. (A) Raman spectra of seven points with an equidistant spacing of 1 cm, starting from Number 1, running top to bottom. (B) Raman spectra of seven points with an equidistant spacing of 1 cm, starting from Number 2, running from left to right. Inset shows an as‐marked LN wafer

From Equations (7) and (8), it can be seen that the linewidth of the characteristic Raman peak of LN decreases continuously under increasing Li content in the LN crystal. To measure the uniformity of the LN wafer's chemical composition, we can plot several equidistant points on the wafer and measure the Raman spectra of these points in sequence (Figure 2). Finally, the lithium content at each point is calculated using the linewidth obtained by fitting (Table 6). The image of the composition and lithium‐content distributions in the LN crystal can be obtained using micro‐Raman imaging technology, and the uniformity of the chemical composition of the LN crystal can be assessed intuitively. In addition, the visualization of the stress and intrinsic defect distributions of LN crystals can also be realized.^[^ ^43^ ^]^ Bartasyte et al.^[^ ^44^ ^]^ utilized 2D mapping of the LN and LiNb_3_O_8_ phase distribution on the surface of a film deposited on R‐sapphire, using Raman spectroscopy. The residual stress distribution and Li stoichiometric uniformity on the substrate surface were assessed using Raman spectroscopy mapping.

**TABLE 6:** Linewidths obtained via Gaussian fitting of the peak at ∼153 cm^−1^ and LN wafer Li content calculated using Equation (7)

Number	FWHM	Li [mol%]
1‐1	14.39	46.21
1‐2	14.38	46.22
1‐3	14.27	46.27
1‐4	14.08	46.36
1‐5	14.14	46.33
1‐6	14.22	46.29
1‐7	14.12	46.34
2‐1	14.33	46.24
2‐2	14.36	46.22
2‐3	14.39	46.21
2‐4	14.08	46.36
2‐5	14.28	46.26
2‐6	14.31	46.25
2‐7	14.21	46.29

Raman spectroscopy is a type of spectroscopic method used to study molecular vibrations and rotations. Its research objects include phonons, electrons, magnons, spinons, and polaritons in solids. Measurements can be performed under high temperatures, high pressures, external electric fields, magnetic fields, and other conditions.^[^ ^45^ ^]^ The properties of elements under high pressure differ from those under normal pressure; thus, it is necessary to re‐study the elements under high pressures. High‐pressure Raman spectroscopy is a powerful tool for studying structure, property, and matter changes under high pressure.^[^ ^46^ ^]^ Dong et al.^[^ ^47^ ^]^ conducted high‐pressure Raman spectroscopy studies on LN in the pressure range of 0–40 GPa. Under an increase in pressure, the frequency of all Raman spectral lines increased linearly, the spectral lines gradually widened, the intensity gradually weakened, and all spectral lines disappeared at 33.2 GPa. The Raman spectra obtained from 33 GPa depressurized LN resembled those of polycrystalline LN, though the lines were much broader than those of polycrystalline LN. However, at a 37 GPa pressure relief, only two broad and weak scattering bands were observed in the Raman spectrum; this differs from the Raman spectra of polycrystalline materials and is very similar to that of LN glass. Currently, high‐temperature synthesis is a widely used production method for various materials. It is of great theoretical and practical value to study the structure of the melt at high temperatures, as well as its reorganization mechanism with respect to temperature change.^[^ ^48^ ^]^ Li et al. studied the structural characteristics of LN crystals at high temperatures using high‐temperature Raman spectroscopy. Under the increase in temperature, the position of each spectral peak moved in the direction of low wavenumber, the number and intensity of spectral peaks decreased, and the spectral peaks widened.^[^ ^49^ ^]^ By measuring and analyzing the Raman spectra of LN crystals at different temperatures, we can study the structural evolution at high temperatures; this provides an experimental basis for further studying the micro mechanisms of LN crystal growth.

## 4 CHARACTERIZATION FOR PHASE AND CHEMICAL STATE

### 4.1 X‐ray diffraction

The x‐ray diffraction (XRD) technique is one of the most important and popular tools used to characterize the structural properties of LN crystals. The most common method of characterizing LN via XRD is to subject the experimentally obtained sample to x‐ray radiation and detect the x‐ray image at a 2*θ* scattering angle; this produces an XRD pattern that can be compared with a standard Powder Diffraction File card to determine the structural characteristics of the sample's phases.^[^ ^50^ ^]^ Taking the different diffraction peaks (corresponding to different crystallographic planes) and calculating the estimated values of the lattice parameters,^[^ ^51^ ^]^ the specific phases and main orientations can be obtained.^[^ ^52^ ^]^

According to the Scherrer formula, the crystallite size of the sample can be calculated as follows

(11) D=Kλβcosθ,

where K is a constant, *λ* is the wavelength of the x‐ray, *β* is the full‐width‐at‐half‐maximum (FWHM) of the XRD pattern, and *θ* is the Bragg's angle.

Bragg's equation,

(12) d=2λsinθ,

can be used to obtain the grain surface spacing. The dislocation density (δ) and strain (ε) of the sample can be determined using^[^ ^53^ ^]^

(13) $$\delta =1/{D^{2}}_{\mathrm{hkl}},$$

(14) ε=β/4tanθ.

The lattice constants, inter‐planar angle, and unit cell volumes can be obtained according to the lattice geometry equation:^[^ ^54^ ^]^

(15) $$\frac{1}{d^{2}}=\frac{4}{3}\left(\frac{h^{2}+hk+k^{2}}{a^{2}}\right)+\frac{l^{2}}{c^{2}}.$$

**FIGURE 3:**
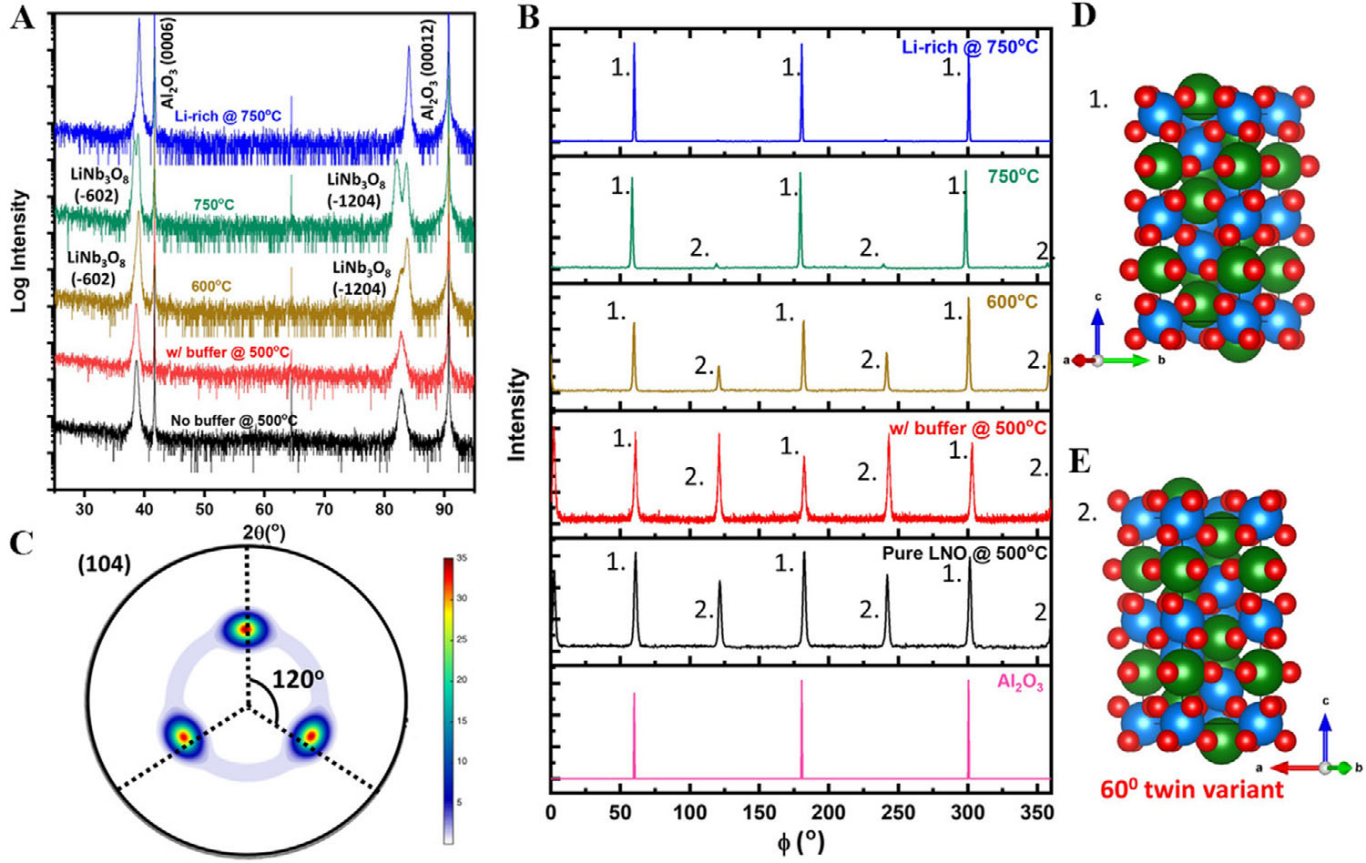
(A) XRD patterns of as‐prepared LN films. (B) In‐plane phi‐scan patterns of different LN films and substrates. Labels 1 and 2 denote variant and normal peaks, respectively. (C) Pole figure at a temperature of 750°C for an Li‐enriched target. Model of LN stacking for (D) normal variant and (E) 60° in‐plane twin variant. Reproduced with permission.^[^ ^58^ ^]^ Copyright 2021, Wiley

Here, *h*, *k*, and *l* are the Miller indices and *a* and *c* are lattice constants. At room temperature, the lattice constants of the LN crystals are *a* = 5.14829 Å and *c* = 13.8631 Å; thus, the cell volume can be solved to determine the variation in the LN lattice under the effects of doping ions,^[^ ^55^ ^]^ and the splitting of the lattice diffraction angle can be used to determine the crystal defects.^[^ ^56^ ^]^ The above data were calculated after the XRD was refined by Rietveld.^[^ ^57^ ^]^ Figure 3 shows the XRD study of the phase and crystal orientations of the as‐grown LN films.^[^ ^58^ ^]^

### 4.2 Neutron diffraction

Neutron diffraction (ND) refers to the Bragg diffraction produced when neutrons (thermal neutrons) with a de Broglie wave of ∼1 Å pass through a crystal substance. Neutrons incident on the crystal scatter at the crystal surface, and an interference effect occurs in the scattered waves, producing a diffraction pattern. ND can more easily identify the occupancy of light elements in LN crystal cells, and its penetrating power is so strong that it is often used in conjunction with XRD to characterize the crystal structures of LN samples.^[^ ^59^ ^]^

### 4.3 X‐ray photoelectron spectroscopy

In X‐ray photoelectron spectroscopy (XPS), the inner or valence electrons of the LN samples are excited by x‐rays; these are referred to as photoelectrons. Photoelectron energy spectra are used to obtain information about the LN samples, by measuring the energy and number of photoelectrons. By analyzing the energy spectrogram, we can obtain the relative content of individual elements in LN samples^[^ ^60^ ^]^ and observe their chemical state via the charge variation of different elements.^[^ ^61^, ^62^ ^]^ Thus, the oxidation state and concentration of Nb, O, and doped atoms in LN crystals can be quantitatively determined.

## 5 MICROSTRUCTURE CHARACTERIZATION

### 5.1 Scanning electron microscopy

Scanning electron microscopy (SEM) is a high‐precision technique used for high‐resolution micro‐area morphology analysis; it can scan LN crystal samples using a focused, narrow, high‐energy electron beam, to extract physical information from the interaction between the electron beam and LN sample; then, it collects, magnifies, and re‐images this information. It has the advantage of a significant depth of field and three‐dimensional imaging capacities. The surface morphologies and grain sizes in LN crystal microstructures can be observed using SEM.^[^ ^63^, ^64^ ^]^ Meanwhile, the elemental composition of the sample can be later analyzed using paired energy dispersive spectroscopy (EDS), to determine whether the experimental sample meets the desired requirements.^[^ ^65^ ^]^ SEM‐EDS combined with XRD mapping can confirm the presence of lattice damage, deformation, and stress in the structure of doped elements,^[^ ^66^ ^]^ and it can observe how the doped elements are distributed in the structure.^[^ ^67^ ^]^ SEM has been widely used to investigate the domain structures of LN ferroelectrics. Since the 1970s, it has also been used to image ferroelectric domains.^[^ ^68^ ^]^ SEM can observe the domain structure without requiring coating or treating of the sample's surface, simply by applying a low‐pressure electron beam.^[^ ^69^, ^70^ ^]^ This makes it possible to compare the distribution of defects near the ferroelectric domain wall region.^[^ ^71^ ^]^ The electron beam can produce potential undulations on the surface of ferroelectric domains in the sample,^[^ ^72^ ^]^ and researchers have therefore used the SEM electron beam for domain writing, a non‐contact method which places no restrictions on the size and period of the domains.^[^ ^73^, ^74^ ^]^ SEM has the advantage of excellent flexibility in the related research and easy integration with other advanced characterization methods; it still retains promising potential in the field of unlocked ferroelectric domain walls.^[^ ^75^ ^]^

### 5.2 Transmission electron microscopy

**FIGURE 4:**
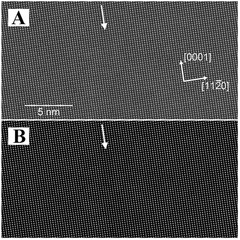
(A) The [1100] HAADF‐STEM image of the ferroelectric domain wall of LN. (B) Fitted model of the HAADF‐STEM image. The arrows denote the direction and location of the LN‐SiC interface. Reproduced with permission.^[^ ^79^ ^]^ Copyright 2016, Wiley

**FIGURE 5:**
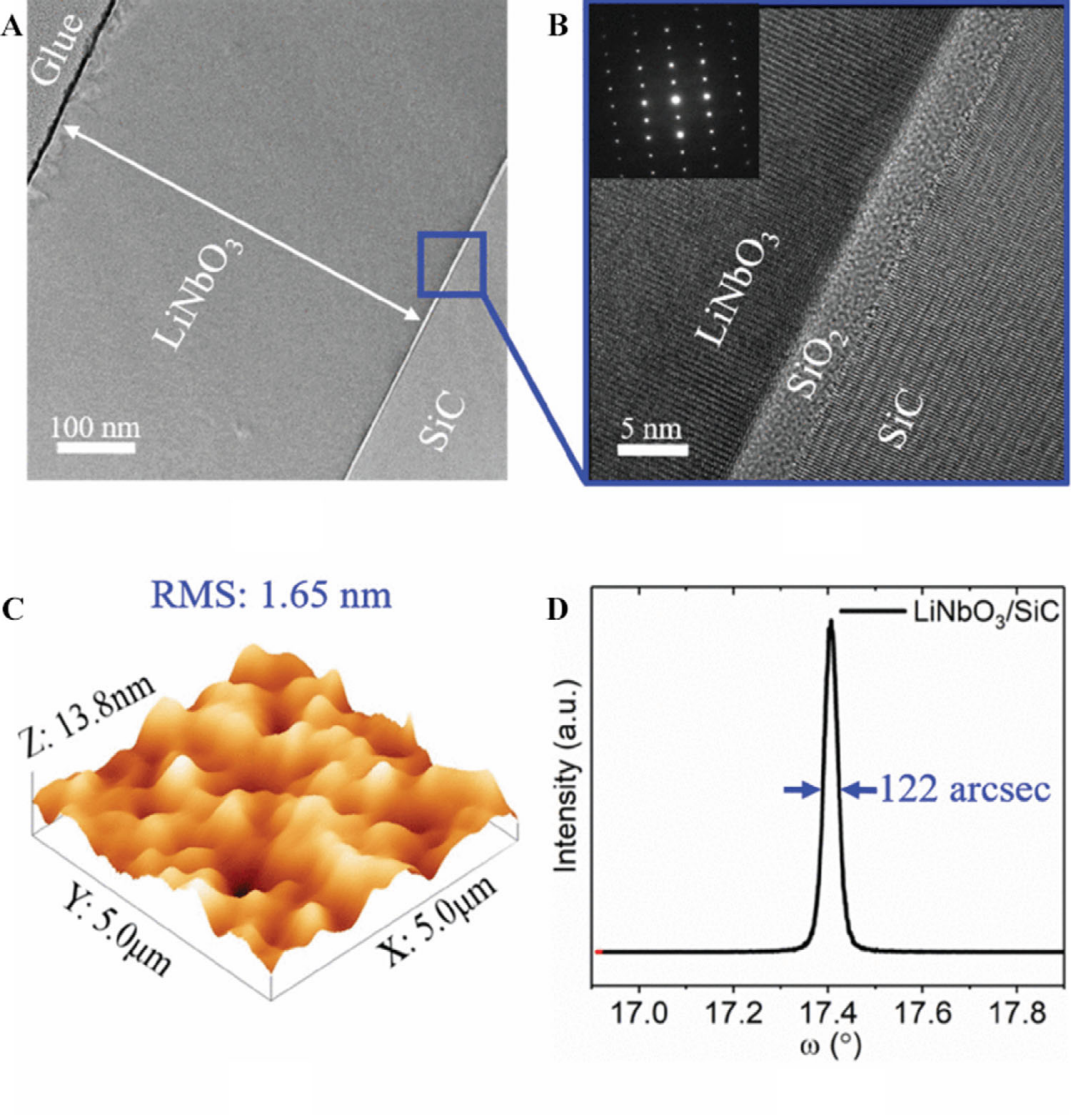
(A) Cross‐section TEM image and (B) HRTEM image of a single‐crystalline LN thin film on a 4H‐SiC substrate. (C) AFM image of the transferred LN thin film after inductively coupled plasma etching. (D) XRD rocking curve for (110) plane of the transferred LN thin film. Reproduced with permission.^[^ ^80^ ^]^ Copyright 2020, IEEE Xplore

Transmission electron microscopy (TEM) typically uses electrons to collide atoms in the sample and produce stereo angular scattering; this is used to image the microstructure. Because of the different structures of the sample, the feedback information also differs, and after electromagnetic imaging, different light and dark images are formed and displayed. In addition to observing the sample morphology and internal structure, TEM can also capture high‐resolution projection images via selected area electron diffraction (SAED). Because of this feature, TEM can be used to observe the morphology and structure of LN nanoparticles^[^ ^67^ ^]^ and calculate the particle size from the measured results.^[^ ^76^ ^]^ The patterns shown by SAED maps can also be used to determine and confirm the morphological characteristics of mechanical deformations such as twinning.^[^ ^77^ ^]^ High‐resolution TEM (HRTEM) has a higher resolution than typical TEM, making it suitable for further observing the crystal's internal structure and lattice arrangement.^[^ ^78^ ^]^ In addition to the typical TEM, scanning transmission electron microscopy (STEM) is also suitable for structure imaging. The light range of typical TEM is a surface, whereas that of STEM is a stepwise sweep followed by information collection. Typically, STEM is used in conjunction with high‐angle annular dark field (HAADF) measurements to obtain information regarding the structure and elemental distribution of micro‐regions of a material. For example, HAADF‐STEM has been used to observe the atomic displacement patterns of ferroelectric domain walls in LN crystals (Figure 4).^[^ ^79^ ^]^ Following the remarkable advances in TEM (especially aberration‐corrected TEM), crystal structure observations can be made at the Angstrom and sub‐Angstrom scales. In recent years, the in situ TEM technique has been used to study the dynamic behavior and atomic structures of multiferroic domains under applied fields or stress.^[^ ^19^ ^]^ This has also helped scholars to further characterize ferroelectric domain structures such as LN. The LN‐SiC interface structure, as imaged by HAADF‐STEM, is shown in Figure 5A,B.^[^ ^80^ ^]^

### 5.3 Atomic force microscopy

Atomic force microscopy (AFM) employs a microcantilever that is extremely sensitive to weak forces at one end; a tiny probe at the other end approaches the sample, and when the distance between the two atoms decreases to a certain level, the interatomic force increases rapidly, causing the microcantilever to deform or change its motion pattern. During scanning of the sample, the sensor is used to detect changes and obtain information regarding the sample surface with a nanoscale resolution. The AFM characterization of LN crystal samples can successfully obtain their surface morphologies^[^ ^81^ ^]^ and roughnesses^[^ ^82^ ^]^ (Figure 5C).

### 5.4 Piezoresponse force microscopy

Piezoresponse force microscopy (PFM) is commonly used for ferroelectric and piezoelectric materials such as LN crystals. The piezoelectric tensor and phase information are obtained by contacting the sample with a needle tip and applying a voltage that causes the sample to expand, or contract and oscillate.^[^ ^83^, ^84^ ^]^ Conductive probe atomic force microscopy (C‐AFM) can extract a conductivity or current map by scanning the current between the conductive tip and sample with a probe grating during contact; this can map the current distribution and surface topography simultaneously; therefore, it is often used in combination with PFM for LN samples.^[^ ^85^ ^]^

### 5.5 Direct piezoelectric force microscopy

Direct piezoelectric force microscopy (DPFM) represents a new technique for directly measuring piezoelectricity; it has made substantial progress in probing the ferroelectricity of halide perovskites, by measuring periodically polarized LN contrasts.^[^ ^86^ ^]^

## 6 PHYSICAL PROPERTY CHARACTERIZATION

### 6.1 Hardness and density

Testing the hardness of LN crystals is essential in precision machining. There are two main methods for testing this hardness: scratch and indentation testing. The principle of scratch hardness is relatively simple, and a representative example is the Mohs hardness. The Mohs hardness of LN crystals has been measured as 5.^[^ ^87^, ^88^ ^]^ The indentation hardness of LN crystals include the Vickers, Knoop, and nanoindentation hardness.^[^ ^89^, ^90^, ^91^ ^]^ Currently, the mainstream hardness test method is the nanoindentation test. The crystal density of LN is usually determined using Archimedes’ principle.^[^ ^92^ ^]^

### 6.2 Electrical resistance

It is difficult to measure the resistance of LN crystals. During testing, the results are affected by the impurities adsorbed on the surface, as well as the interference of pyroelectric signals. The principle of resistance measurement for LN crystal resembles that of conventional resistance measurement, which uses a two‐electrode system to apply a DC voltage to the LN sample for accurate measurement.^[^ ^93^, ^94^, ^95^ ^]^

### 6.3 Refractive index

LN is an essential integrated photonics platform, and the measurement of its refractive index is necessary for the development and production of optical unit devices.^[^ ^96^ ^]^ There are many methods for measuring the refractive index of LN crystals, including the prism coupler method^[^ ^97^, ^98^, ^99^ ^]^ and the minimum angle of deviation method;^[^ ^100^ ^]^ an UV–vis spectrophotometer can also be used to measure the absorption spectrum and thereafter calculate the refractive index.^[^ ^97^, ^101^ ^]^ The minimum angle of deviation method requires the sample to be processed into a prism, which is unsuitable for measuring the refractive index of optical devices. The more commonly used UV–vis absorption spectroscopy and prism‐coupling methods place fewer requirements upon sample processing and are more convenient for testing.

### 6.4 Electro‐optic coefficient

When a beam of light enters a crystal and propagates inside, the refractive index varies with the direction of propagation and polarization. The anisotropy of light propagating in the crystal is not only determined by the internal structure of the crystal but also by the change in the refractive index, which can be realized by external induction. The phenomenon by which the optical properties of a crystal are changed under application of a DC electric field is called the electro‐optical effect. Crystals exhibiting an electro‐optic effect are called electro‐optic crystals. LN crystals have excellent electro‐optic properties and can be used to develop various electro‐optic devices. At present, six methods are primarily used to measure the electro‐optic coefficient of electro‐optic crystals; these include ellipsometry, the half‐wave voltage method, interferometry, and the single‐path MR interferometer method.^[^ ^102^, ^103^, ^104^ ^]^ Wang et al. determined the electro‐optical coefficient and its dispersion for LN, using photoelastic modulation. The spectral polarization method was constructed using a photoelastic modulator, and a monochromator was used to scan the light source wavelength. The phase delay caused by the photoelectric sample was loaded into the modulation signal, to demodulate the photoelectric coefficient.^[^ ^105^ ^]^ Li et al. used a photoelastic modulator to measure electro‐optic coefficients. Using digital phase‐locking technology, the DC and the first and second harmonic terms were extracted separately; then, the electro‐optic coefficients were demodulated.^[^ ^106^ ^]^ Zhang et al. used prism‐coupling technology to measure the photoelectric coefficient of LN simply, quickly, and conveniently. This test method is not only applicable to bulk crystalline materials but also to thin‐film or planar/slab waveguide structures.^[^ ^107^, ^108^ ^]^

### 6.5 Thickness

**FIGURE 6:**
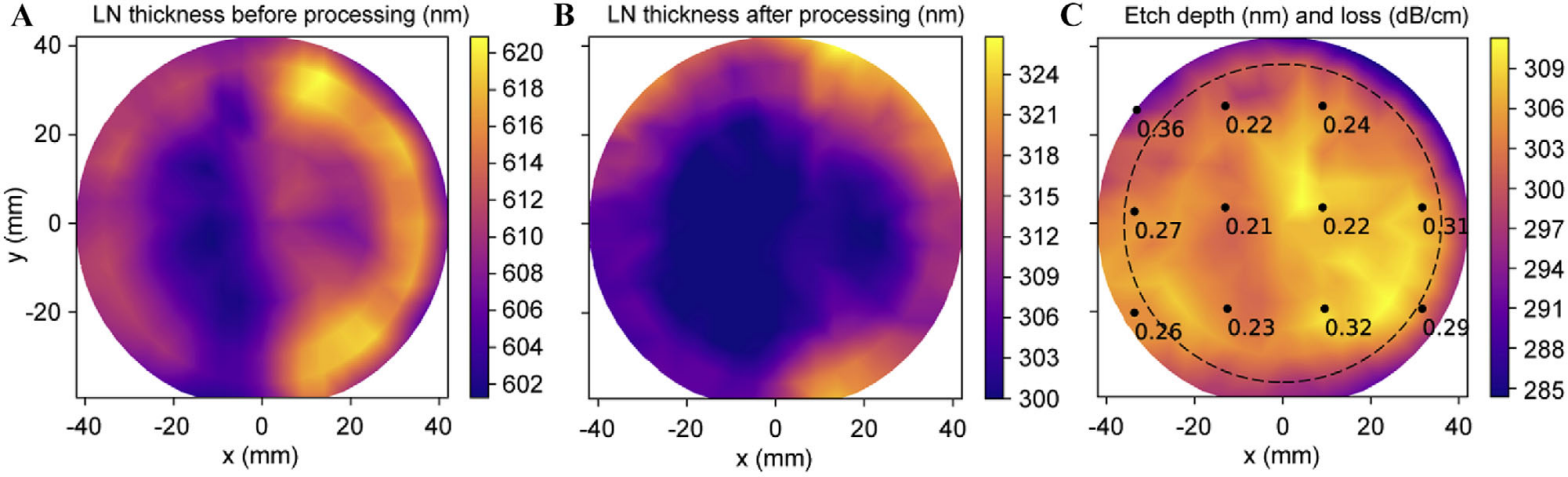
Four‐inch LN wafer thickness uniformity characterization: (A) before device processing and (B) after device processing. (C) Etch depth difference mapping between (A) and (B). Reproduced with permission.^[^ ^109^ ^]^ Copyright 2020, The Optical Society

The thickness measurement of large‐size LN wafers (e.g., 4‐ and 6‐inch) is important for high‐throughput wafer‐scale processing. The wafer‐scale film thickness can be measured with an interferometer.^[^ ^98^ ^]^ Because of the relatively large light spot size and roughened rim on LN wafers, the 8‐mm edge of the 4‐inch wafer could not be measured (Figure 6A).^[^ ^109^ ^]^ After deep ultraviolet lithography processing, the etch depth standard deviations were 5.9 nm across the wafer and 3.2 nm within the dotted circle (Figure 6B,C). More importantly, the etch depth variation agreed with the thickness variation of the initial LN wafer.

### 6.6 Interface electric field

**FIGURE 7:**
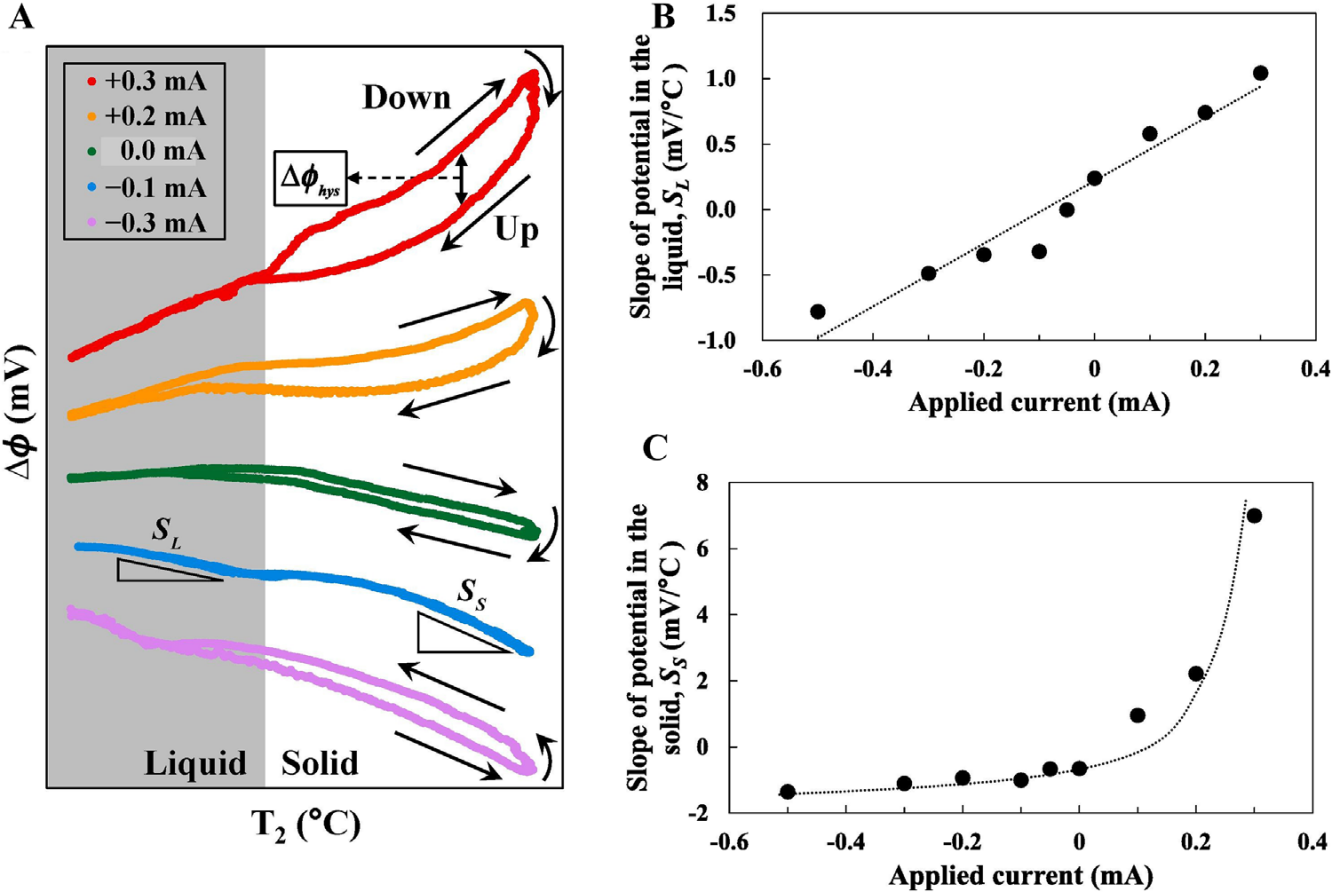
Measurement of interface electric field at melt and crystal interface of Mg: LN. (A) Δϕ‐*T*2 curve during growth and melting process. The slopes of the Δϕ‐*T*2 curves with respect to applied current in (B) LN melt and (C) LN crystal. Reproduced with permission.^[^ ^112^ ^]^ Copyright 2020, Elsevier

At the LN's solid–liquid interface, a high‐temperature gradient can result in the Seebeck effect, which can form an intrinsic interfacial electric field. Within the LN melt, ionic species (e.g., Li^+^, LiO^−^, Nb_2_O_4_V_O_^2+^, and O^2−^) can be found.^[^ ^99^ ^]^ During LN crystallization from the melt, the formed interface electric field can control the transport and partitioning of ionic species.^[^ ^110^, ^111^ ^]^ Recently, the partitioning effect of ionic species upon the interface electric field has been studied (Figure 7).^[^ ^112^ ^]^ The results showed that all particle species coefficients in the melt are 1 for congruent LN, and a Schottky contact between the LN melt and LN crystal is formed. In addition, the unsteady crystal growth of LN in the presence of an interfacial electric field has also been studied.^[^ ^113^ ^]^ When the equilibrium partition coefficient was not equal to 1, the sudden growth rate change produces a transient increase or decrease in the melt solute concentration near the interface. The results showed that the intrinsic electric field could be balanced during the crystal growth process by injecting an external current into the solid–liquid interface. The equilibrium partition coefficient of each ion species was 1, indicating good compositional uniformity in both the solid and liquid phases. The interface electric field can serve as an in situ method to elucidate the change in the solid–liquid interface during crystal growth. Zhu et al. used the growth interface electromotive force to identify interface fluctuations, reveal convection instabilities, and visualize the LN growth rate and temperature fluctuations.^[^ ^114^, ^115^, ^116^ ^]^

### 6.7 Optical waveguide propagation loss

LN thin films have the advantages of a wide transmission window, strong electro‐optical nonlinearity, and compact properties; they are expected to become the materials of choice for realizing multifunctional, high‐performance integrated photonic circuits for classical and quantum applications platforms.^[^ ^117^, ^118^ ^]^ The optical waveguide is an essential component in the integrated optical system, and the transmission loss of the waveguide is a critical parameter of the integrated optical device.^[^ ^119^ ^]^ For resonant structures and large‐scale photonic integrated circuits, waveguide losses directly affect performances.^[^ ^120^ ^]^ The waveguide loss measurement methods of the integrated optical waveguide fabricated on the substrate include the waveguide‐scattered light intensity method, the pyroelectric effect method, calorimetry, the prism method, and the Fabry–Perot resonant cavity method.^[^ ^121^, ^122^ ^]^ Fabry–Perot interferometry is performed by measuring the change of the contrast in the Fabry–Perot cavity with respect to the length of cavity; it is commonly used to measure the propagation loss.^[^ ^123^ ^]^ Improved Fabry–Perot methods are also available; for example, the loss of the waveguide can be obtained by analyzing the fineness of the reflection interference pattern for a Fabry–Perot cavity formed by a single‐mode waveguide facet.^[^ ^120^ ^]^ Sattibabu et al.^[^ ^124^ ^]^ proposed a non‐destructive and simple spectroscopic method to measure a single‐mode optical waveguide's coupling and transmission losses, and they demonstrated this technique using a fabricated Ti:LN waveguide. Santandrea et al.^[^ ^125^ ^]^ introduced a method to estimate the loss of nonlinear waveguides at multimode wavelengths. By fitting the phase‐matched spectra of different titanium‐diffused LN waveguides to their model, a single spatial second‐harmonic loss of the mode at the spatial multimode wavelength was obtained.

## 7 CONCLUSIONS AND OUTLINE

During the crystal growth stage, the composition, phases, morphology, defects, and microstructures of LN raw materials, melts, and crystals require numerous measurement technologies, to satisfy the practical demands of crystal quality; these technologies can test multi‐scale LN samples ranging from nanometers to bulk centimeters, and at different temperatures ranging from room temperature to melting point (more than 1200°C). Advanced aberration‐corrected TEM, spectrum mapping, and acoustic methods have been successfully used to characterize LN wafers and films.

LN in the form of wafers and single‐crystal films has been widely used for various applications in acoustic, optical, and optoelectronic devices. Therefore, the physical and chemical properties, thicknesses, and crystallographic orientations (cutting orientations) of LN crystals require accurate measurements to determine their quality and application fields (e.g., acoustic and optical devices). For LN quality control and product testing procedures, fast and convenient measurement methods are in high demand.

Following instrumentation development, measurement methods with higher spatial and temporal resolutions have helped us to acquire more detailed information regarding LN crystals (e.g., point defects, domain structures, dynamics, and quantum mechanics). However, conventional testing technologies still have enormous potential to reveal the composition and microstructures of LN melts and crystals. For future applications, interdisciplinary studies of LN crystals, wafers, and devices are urgently needed to promote the further development of LN crystals.

## CONFLICT OF INTEREST

The authors declare no conflict of interest.
